# Murine and Human Cathelicidins Contribute Differently to Hallmarks of Mastitis Induced by Pathogenic *Prototheca bovis* Algae

**DOI:** 10.3389/fcimb.2020.00031

**Published:** 2020-02-07

**Authors:** Muhammad Shahid, Paloma Araujo Cavalcante, Cameron G. Knight, Herman W. Barkema, Bo Han, Jian Gao, Eduardo R. Cobo

**Affiliations:** ^1^Department of Clinical Veterinary Medicine, College of Veterinary Medicine, China Agricultural University, Beijing, China; ^2^Department of Production Animal Health, Faculty of Veterinary Medicine, University of Calgary, Calgary, AB, Canada; ^3^Department of Veterinary Clinical and Diagnostic Sciences, Faculty of Veterinary Medicine, University of Calgary, Calgary, AB, Canada

**Keywords:** *Cramp*, LL-37, *Prototheca bovis*, mastitis, murine model

## Abstract

*Prototheca bovis* (formerly *P. zopfii* genotype-II) is an opportunistic, achlorophyllous alga that causes mastitis in cows and skin disease in cats and dogs, as well as cutaneous lesions in both immunocompetent and immunosuppressed humans. Antifungal medications are commonly ineffective. This study aimed to investigate innate immune responses contributed by cathelicidins to *P. bovis* in the mammary gland using a mastitis model in mice deficient in the sole murine cathelicidin (*Camp*). We determined *P. bovis* caused acute mastitis in mice and induced *Camp* gene transcription. Whereas, *Camp*^−/−^ and *Camp*^+/+^ littermates had similar local algae burden, *Camp*^+/+^ mice produced more pro-inflammatory cytokines, TNF-α, and Cxcl-1. Likewise, *Camp*^+/+^ bone marrow-derived macrophages were more responsive to *P. bovis*, producing more *TNF-*α and *Cxcl-1*. Human cathelicidin (LL-37) exhibited a different effect against *P. bovis*; it had direct algicidal activity against *P. bovis* and lowered *TNF-*α, Cxcl-1, and *IL-1*β production in both cultured murine macrophages and mammary epithelial cells exposed to the pathogenic algae. In conclusion, cathelicidins were involved in protothecosis pathogenesis, with unique roles among the diverse peptide family. Whereas, endogenous cathelicidin (*Camp*) was key in mammary gland innate defense against *P. bovis*, human LL-37 had algicidal and immunomodulatory functions.

## Introduction

*Prototheca* species are unicellular achlorophyllous algae, 3–30 μm in diameter, that lack a specific glucosamine cell wall or chloroplasts (Baudelet et al., 2017). Their reproduction is asexual, with endospores being released from sporangia (Jagielski and Lagneau, 2007). *Prototheca* spp. are widely distributed in the environment, particularly in organic matter with a high moisture content (Scaccabarozzi et al., 2008). *Prototheca* spp. are also pathogenic and provoke a variety of maladies in animals. In dogs and cats, infection with *Prototheca* spp. causes either cutaneous lesions or systemic disease with hemorrhagic enteritis and progressive retinal degradation (Huth et al., 2015; Carfora et al., 2017). In humans, *Prototheca* spp. causes rare chronic skin or articular infections (Seok et al., 2013) or disseminated infections mostly in immunodeficient patients (Tello-Zavala et al., 2013).

Among Prototheca species, *P. bovis* has been proposed as a major cause of chronic mastitis in cattle. Traditional taxonomic studies on Prototheca species identified two genotypes (GT): *P. zopfii* GT- I and *P. bovis* (formerly *P. zopfii* GT-II) (Roesler et al., 2006). *P. bovis* was isolated from the milk of cows with mastitis and identified as the causative agent of bovine mastitis (Jagielski et al., 2012, 2019a,b,c,d; Bozzo et al., 2014). *Prototheca zopfii* GT-I is commonly isolated from the environment and occasionally causes granulomatous lesions in experimentally infected bovine udders (Ito et al., 2011) and protothecosis in humans (Hirose et al., 2018). Clinical signs of protothecal mastitis in cattle can include fever, pain, edema, anorexia, and lethargy, although it is most commonly subclinical, causing decreased milk production (Wawron et al., 2013). Bovine mastitis caused by *P. bovis* is refractory to most therapeutic agents (Jagielski et al., 2012) and has a very low rate of spontaneous remission (Janosi et al., 2001). Virulence factors, including intracellular heat shock proteins (Hsp70) and proteins that augment peroxisome metabolism to resist harsh environments (e.g., macrophage phagolysosomes) are mostly present in pathogenic *P. bovis* (Irrgang et al., 2015; Zeng et al., 2019). From an epidemiological perspective, cattle infected with *Prototheca* spp. are a common source of *Prototheca* spp. for humans, with immunocompromised farmers at highest risk (Lass-Florl and Mayr, 2007). Since *Prototheca* spp. may survive chlorination by forming biofilms (Kwiecinski, 2015), and be returned to the environment via sewage effluent and household waste (Wirth et al., 1999), cows with mastitis caused by *P. bovis* are a risk factor in epidemiology of protothecosis (Chao et al., 2002).

Little is known about innate mammary gland defenses during *P. bovis* infection. However, cultured mammary epithelial cells from cattle and mice undergo oxidative stress and apoptosis when exposed to *P. bovis* (Shahid et al., 2016, 2017a,b). Moreover, bovine mammary epithelial cells respond to *P. bovis* infection with upregulated expression of Toll Like Receptors (TLRs)−2 and−4, pro-inflammatory cytokines (TNF-α, IL-1β, IL-8) and β-defensin-5 (Deng et al., 2016). To further understand innate mammary defenses in protothecosis, we focused on cathelicidins. Cathelicidins are cationic amphipathic peptides with an N-terminal domain containing a signal peptide, a well-conserved central cathelin domain, and a variable C-terminal domain (Schauber et al., 2003). The C- terminal domain is cleaved to produce the peptide with antimicrobial and antifungal activity (Ordonez et al., 2014). In humans, a single cathelicidin gene (*cathelicidin antimicrobial peptid*e, *Camp*) yields this C-terminal active peptide termed leucine-leucine with 37 amino acid residues (LL-37) (Agerberth et al., 1995). In mice, the functional homologous peptide is cathelicidin-related-antimicrobial-peptide (Cramp) encoded by the gene *Camp* (Nizet et al., 2001). Cathelicidins are abundantly expressed in mammalian cells, mainly neutrophils and epithelial cells and thus, are present in various organs/tissues, including skin, eyes, mouth, lungs, intestine, and mammary gland (Zanetti, 2005; Cobo et al., 2012, 2017). Cathelicidins have been involved in various host responses against bacterial and parasitic intracellular pathogens through activation of cytokines/chemokine secretion (Boucher et al., 2018; Cirone et al., 2019; Marin et al., 2019). In sheep and murine mammary glands and in human milk, cathelicidins also have antimicrobial and anti-inflammatory effects (Murakami et al., 2005; Addis et al., 2013). Moreover, cathelicidins increased in bovine mammary epithelial cells exposed to a main mastitis pathogen, *Staphylococcus aureus* (Ibeagha-Awemu et al., 2010). Thus, we hypothesized that cathelicidins produced by the mammary gland contribute to host innate defense against *P. bovis* infection. Using genetically mutant mice that lack cathelicidin (*Camp*^−/−^) and cultured murine macrophages and mammary epithelial cells, we demonstrated that endogenous cathelicidins regulate mammary gland inflammation and macrophage activity against *P. bovis*, whereas exogenous human cathelicidins (LL-37) downregulated epithelial and macrophage responses and had algicidal activities.

## Materials and Methods

### Ethics Statement

Animal experiments were conducted in accordance with Canadian Guidelines for Animal Welfare (CGAW) and the University of Calgary Animal Care Committee (Animal Protocol AC16-0061).

### Prototheca bovis

A *Prototheca* spp. was isolated from the milk of a cow with clinical mastitis at the College of Veterinary Medicine, China Agricultural University, Beijing, China (Gao et al., 2012). This *Prototheca* spp. was characterized as *P. bovis* by several methods. First, we characterized its cellular fatty acid pattern to confirm that *P. bovis* had, as expected, more eicosadienoic acid (C20: 2) compared to the reference *P. zopfii* GT-I (Roesler et al., 2006). Second, we determined 18S rDNA sequences using genotype-specific PCR (Roesler et al., 2006; Moller et al., 2007). For this, *P. bovis* was purified from a single colony of *P. bovis* isolated from Sabouraud dextrose agar (Sigma-Aldrich) and amplified in Sabouraud in dextrose broth (Sigma-Aldrich) (1 mL) (DP302, TIANamp DNA, Tiangen). DNA was quantified (NanoDrop ND-1000 Spectrophotometer, Thermo Fisher Scientific) and stored at −20°C. The *Prototheca* (450 bp) fragment internal amplification control was detected using *Proto18-4f* (GACATGGCGAGGATTGACAGA) and *Proto18-4r* (AGCACACCCAATCGGTAGGA) sequences. The *P. bovis* specific amplicon (165 bp) was detected with primers *Proto18-4f* (GACATGGCGAGGATTGACAGA) and *PZGT-II/r* (GTCGGCGGGGCAAAAGC) (Roesler et al., 2006; Moller et al., 2007).

The *P. bovis* genotype was further confirmed by restriction fragment length polymorphism (RFLP) analysis targeting the *cytb* gene fragment (599–668 bp) (Jagielski et al., 2018). For this, a PCR mix (25 μL) containing cytb-F1 (5′ GyGTwGAACAyATTATGAGAG-3′) and cytb-R2 (5′-wACCCATAArAArTACCATTCwGG-3′) primers (10 μM each primer), DNA template (1 μL), and 2x EasyTaq PCR supermix (TransGen Biotech, AS111-11; 12.5 μL) was amplified under specific conditions (2 min at 95°C, followed by 35 cycles of 30 s at 95°C, 30 s at 50°C, and 30 s at 72°C, with final extension of 5 min at 72°C). The PCR products depicted a 644 base pair (bp) product compatible with *P. zopfii* as visualized by agarose gel electrophoresis (1%, wt/vol) and stained with ethidium bromide (Supplementary Figure 1A). The amplified *cytb* gene products (644-bp) were digested by RsaI and TaiI digesting enzymes (FastDigest Enzymes, Thermo Fisher Scientific). The total mixture (30 μL) containing 10x restriction enzyme buffer (3 μL), PCR product (10 μL), enzymes (1.5 μL each) and PCR water (16.5 μL) was digested by RsaI (5 min at 37°C) followed by TaiI (5 min at 65°C). The restriction products visualized on 4% agarose gels, stained with ethidium bromide, and exposed to UV light showed DNA fragments of 200 and 450 bp after RSaI/TaiI digestion, compatible with *P. bovis* (Supplementary Figure 1B). Taken together, we confirmed a *P. bovis* genotype in the isolate clinically recovered from a case of mastitis in cows.

For experimentation, *P. bovis* was streaked on Sabouraud dextrose agar (S3181, Sigma-Aldrich) (37°C for up to 48 h) to produce typical single creamy-white, yeast-like colonies and replicated in Sabouraud dextrose broth (S3306, Sigma-Aldrich) (37°C for up to 72 h) (Gao et al., 2012).

### Experimental Induction of Murine Mastitis by *Prototheca bovis*

C57BL/6 (6–8 wk. old) lactating female wild-type *Camp*^+/+^ and cathelicidin-null *Camp*^−/−^ C57BL/6 mice (B6.129X1-*Camp*^tm1Rlg/J^; The Jackson Laboratory) were housed in a specific pathogen-free environment with *ad libitum* access to feed and water (University of Calgary). In these *Camp*^−/−^ mice, there is deletion of exons 3 and 4 of the cathelicidin gene *Camp*; whereas some gene portions could be detected, they do not produce functional cathelicidin peptides (Nizet et al., 2001). *Camp*^−/−^ and *Camp*^+/+^ mice were infected intramammary (10–14 days after parturition) with either *P. bovis* (50 μL containing 1 × 10^5^ colony forming units (CFU)/mL) or an equal volume of phosphate buffered saline (PBS) (control) in the left 4th and right 4th mammary glands (L4 and R4) (n: 4 per group). Mice were euthanized by carbon dioxide followed by cervical dislocation at 4 days post-infection when they had indications of clinical mastitis. This termination point was chosen based on preliminary studies where mice become lethargic and before humane end points associated with longer intervals.

Immediately after euthanasia, all mammary glands were excised, weighed, and collected as follows: A portion was incubated into Trizol (Invitrogen) for gene quantification and another fixed in formalin (10%) solution, embedded in paraffin wax, sectioning (5 μm) and stained with hematoxylin and eosin (H&E) or toluidine blue for histological examination (Seok et al., 2013). Prototheca organisms were identified by specific staining, using periodic acid-Schiff (PAS) and Grocott methenamine silver (GMS).

### Identification of Macrophages and Neutrophils in Murine Mammary Glands

Fixed mammary gland sections were deparaffinized and dehydrated, permeabilized with PBS/Triton X-100 (0.25%, v/v) (PBS-T) buffer with donkey serum (1%; 017-000-121, Jackson ImmunoResearch) [room temperature (RT), 10 min]. Slides were blocked with PBS-T containing donkey serum [10% (v/v)] and bovine serum albumin (BSA) (1% (v/v); CA97061-416, Sigma-Aldrich) (RT, 2 h). After washing with PBS, sections were incubated with primary antibodies against murine F4/80 (macrophages) (4316835, BD Pharmingen) and Ly-6G (neutrophils) antigens (127609, Biolegend) (1:1,000 in PBS-T plus 1% BSA) for 16 h at 4°C. Following washing with PBS-T, slides were incubated with secondary antibodies (Alexa Fluor® 488-conjugated affinipure goat anti-rat IgG, 135205, Jackson Immune Research) (1:1,000 in PBS-T plus 1% BSA) (RT, 1 h), washed again with PBS-T and incubated with DAPI (4′, 6-diamidino-2- phenylindole) (Invitrogen) (RT, 20 min). Slides were examined with an immunofluorescence microscope (Axio Imager M2, Zeiss).

### Determination of *Prototheca bovis* in Infected Mammary Tissue

*Prototheca* infection in mammary tissues was assessed by histological counting of *Prototheca* sporangia and *Prototheca* culture. For counting, sporongia of *Prototheca* spp. (7–30 μM in diameter) were counted in 10 images of the mammary gland per mouse, individually and randomly captured at 40x magnifications. For culturing, the entire mammary gland was homogenized in sterile tubes containing 1 mL of 0.025% Triton X-100 in PBS. 10-fold homogenate dilutions were spread onto duplicate plates of Sabouraud dextrose agar (Sigma-Aldrich). Number of colonies, typically with a granular serrated shape, grayish white and with a central protrusion, was determined after 2–3 days of incubation (Gogoi-Tiwari et al., 2017).

### *P. bovis* Infection in Cultured Murine Bone Marrow-Derived Macrophages and Immortalized Mammary Epithelial Cells and Macrophages

Bone marrow-derived macrophages (BMDMs) were collected from femurs of *Camp*^−/^^−^ and *Camp*^+/+^ mice and plated into RPMI containing 10% fetal bovine serum (Hyclone), macrophage colony stimulating factor (M-CSF, Peprotech) or granulocyte-macrophage colony-stimulating factor (GM-CSF, Peprotech) (10 ng/mL) and antibiotic mixture (penicillin-streptomycin, 1%, P4333, Sigma-Aldrich). BMDMs were cultivated for 7–10 days in 12 well plates and challenged with *P. bovis* (1 × 10^5^ CFU/mL re-suspended in RPMI) (37°C with 5% CO_2_) for up to 8 h. Challenge doses of *P. bovis* were chosen based on previous *in vitro* experiments with bovine mammary epithelial cells infected with *P. bovis* (Shahid et al., 2017b) and a murine model of *P. bovis* mastitis (Shahid, personal communication).

Mammary epithelial and macrophage responses, relevant during mastitis (Lam et al., 2009; Xiao et al., 2015), were modeled using the murine mammary epithelial cell line (HC11), a prolactin responsive clone COMMA-1D derived from mammary tissue of BALB/c mice in mis-pregnancy (Katz and Streuli, 2007); and a murine phagocytic monocyte J774A.1 (ATCC® TIB-67™) derived from BALB/c mice, conventionally used as a macrophage model. To assess the role of exogenous cathelicidin in protothecosis, murine macrophages and mammary epithelial cells were challenged with *P. bovis* (1 × 10^5^ CFU/mL resuspended in DMEM/F12) ± synthetic human cathelicidin LL-37 amide (H-Leu-Leu-Gly-Asp-Phe-Phe-Arg-Lys-Ser-Lys-Glu-Lys-Ile-Gly-Lys-Glu-Phe-Lys-Arg-Ile-Val-Gln-Arg-Ile-Lys-Asp-Phe-Leu-Arg-Asn-Leu-Val-Pro-Arg-Thr-Glu-Ser-NH2 trifluoroacetate salt) (10 μg/mL; H-6224; Bachem) for up to 8 h (37°C with 5% CO_2_).

### Transcriptional Expression of TNF-α, IL-1β and Cxcl-1, and Secretion of Cxcl-1 and TNF-α in Mammary Tissues and Cultured Cells

Gene mRNA transcription was quantified by quantitative real-time polymerase chain reaction (RT qPCR). Total RNA from murine mammary glands, BMDMs, mammary epithelial cells and macrophages was extracted with Trizol reagent (Invitrogen) and converted into cDNA by reverse transcription (101414-098, VWR). The RNA and cDNA quality was evaluated by the A260/A280 absorbance ratio (NanoVue Spectrophotometer, GE Healthcare Bio-Sciences) (Bustin et al., 2009). The pre-designed primers (RT^2^ qPCR Primer Assay, Qiagen) were specific for murine tumor necrosis factor alpha (*TNF-*α) (PPM03113G), interleukin 1 beta (*IL-1*β) (PPM03109F), C-X-C motif chemokine ligand 1 (*Cxcl-1*) (PPM03058C), cathelicidin (*Camp*) (PPM25023A), defensin-4 (PPM29171A) and housekeeping gene *glyceraldehyde-3-phosphate dehydrogenase* (*GAPDH*) (forward 5′ AAATGGTGAAGGTCGGTGTG and reverse 3′ TGAAGGGGTCGTTGATGG). All primers were verified for specificity and efficiency (>95%) to ensure amplification of a single product of the correct size, as indicated in MIQE guidelines (Bustin et al., 2009). The total reaction mixture (10 μL) included 2 μL of cDNA, 0.5 μM of forward and reverse primers and 1x SsoAdvanced Universal SYBR Green Supermix (BioRad) (CFX-96 real-time PCR system). All reactions were performed in triplicate. Target gene mRNA values were corrected relative to the normaliser, *GAPDH*. Data were analyzed using the 2^−ΔΔCT^ method and reported as mean fold change of target transcript levels in challenged vs. uninfected control groups.

### Secreted Cxcl-1 and TNF-α Protein Quantification

TNF-α and Cxcl-1 proteins produced and secreted by the mammary gland were quantified by respective ELISAs (DY453-05 and DY410-05, R&D Systems) in supernatants collected from the whole murine mammary gland homogenized with 1 mL of 0.025% Triton X-100 in PBS and centrifuged (20,000 *g*; 10 min).

### Direct Algicidal Activity of Cathelicidin Against *P. bovis*

For killing assays, *P. bovis* grown into Sabouraud in dextrose broth (Sigma-Aldrich) (37°C, 2 days on a rotary shaker) was poured into 96 wells plates (1 × 10^5^ CFU/mL in logarithmic growth phase) and simultaneously incubated with LL-37 peptides (H-6224; Bachem) (1 and 2 μM diluted in distilled water) (37°C, up to 1 day). Final *Prototheca* spp. concentration was determined by CFU/mL and plotted after conversion to logarithm (log_10_) using a standard curve. *P. bovis* and media only were used as controls.

### Statistical Analyses

Normality was assessed using D'Agostino & Pearson omnibus normality or Shapiro-Wilk (Royston) tests. Analytical data represented as histograms were recorded as mean values with bars representing standard errors of the mean (SEM) from a minimum of two independent experiments, with data obtained in triplicate, unless otherwise stated. All statistical comparisons were performed using one-way analysis of variance (ANOVA) with a *post hoc* Bonferroni correction for multiple group comparisons (Graph Pad Prism, 5.0). A *p-* value was assigned to each experimental group with reference to a control group. A *p-*value of <0.05 was considered significant.

## Results

### *P. bovis*-Challenged Camp^−/−^ Mice Developed More Acute Mastitis With Predominantly Histiocytic Infiltration Compared With Camp^+/+^ Mice

**Figure 1:**
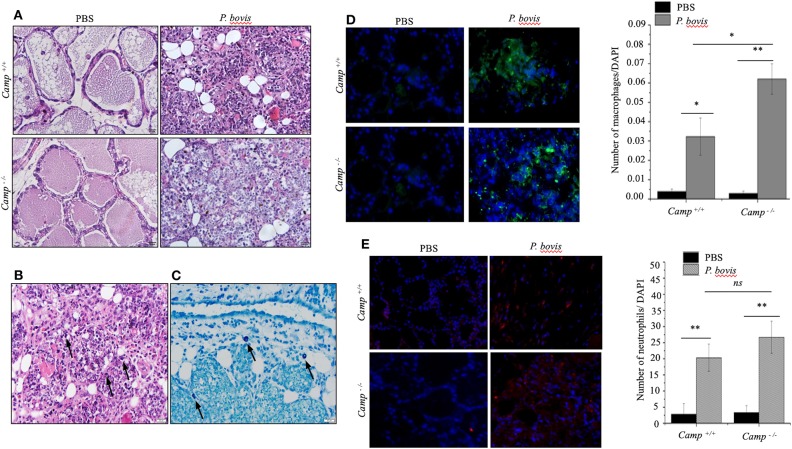
*Prototheca bovis* induced acute mastitis with higher leukocyte infiltration in *Camp*^−/−^ mice. **(A,B)** *Camp*^+/+^ and *Camp*^−/^^−^ mice were challenged intramammary with *P. bovis* or PBS (control). Mammary glands obtained 4 d post-infection were stained with hematoxylin and eosin. **(B)** Mammary gland from *P. bovis* infected *Camp*^+/+^ mouse. Note mammary epithelial lobules, interstitial fibrovascular tissue and interlobular adipose tissue infiltrated by a mixed population of inflammatory cells that includes lymphocytes, plasma cells, neutrophils, macrophages, and rare mast cells. *Prototheca* organisms (black arrows) are oval, approximately 10 μm in length and surrounded by a clear halo. Scale bar = 20 μm. **(C)** Mammary gland of an infected *Camp*^+/+^ mouse stained by toluidine blue for identification of rare mast cells (black arrows) in addition to the numerous mixed inflammatory cells shown in **(B)**. Mast cell cytoplasmic granules highlighted by toluidine blue stain. Scale bar = 20 μm. **(D,E)** Mammary gland from *P. bovis* infected *Camp*^+/+^ and *Camp*^−/^^−^ mice immunostained with anti-macrophage (F4/80) and anti-neutrophil (Ly6G) antibodies. Histograms represent the average number of cells per field (a minimum of 10 randomly chosen microscopic fields, x40 objective magnification). Data are shown as means ± SEM (*n* = 4/group). *p < 0.05, **p < 0.01 (one-way ANOVA *post hoc* Bonferroni correction for multiple group comparison) was considered significant.

Effects of endogenous cathelicidins in mastitis were studied in lactating *Camp*^+/+^ and *Camp*^−/−^ mice intramammary infected with *P. bovis* isolated from a bovine clinical mastitis case. Both *Camp*^+/+^ and *Camp*^−/−^ infected mice developed clinical mastitis (painful, red, and edematous mammary glands) 4 days post-infection, with onset of depression and lethargy. Microscopically, uninfected control mice had normal mammary gland architecture whereas both *Camp*^+/+^ and *Camp*^−/−^ infected mice developed severe acute mastitis (Figure 1A). Mammary epithelial lobules, interstitial fibrovascular tissue and interlobular adipose tissue in both *Camp*^+/+^ and *Camp*^−/−^ infected mice had infiltration of a mixed population of inflammatory cells, including lymphocytes, plasma cells, neutrophils, macrophages (Figures 1A,B) and rare mast cells (Figure 1C). More macrophages infiltrated the mammary in *P. bovis* infected *Camp*^−/−^ mice **(***p* < 0.05, Figure 1D). Neutrophils were numerous in *P. bovis* infected mice, but there was no difference between *Camp*^−/−^ and *Camp*^+/+^ mice (*p* > 0.05, Figure 1E).

**Figure 2:**
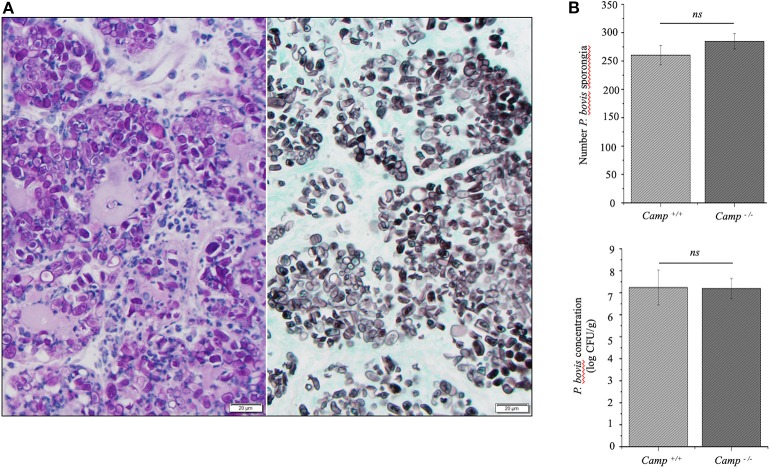
*Prototheca bovis* was present at similar levels in the mammary glands of *Camp*^+/+^ and *Camp*^−/−^ mice. **(A)** Mammary gland from a *Camp*^+/+^ mouse infected with *P. bovis* (4 d post-infection). Abundance of *Prototheca* organisms within tissue sections was revealed with special stains. Prototheca are stained magenta by periodic acid-Schiff (PAS) stain (left image) and black by Grocott methenamine silver (GMS) stain (right image). Scale bar = 20 μm. **(B)** *P. bovis* was present in mammary glands at similar levels in *Camp*^+/+^ and *Camp*^−/−^ mice, as determined by counting of sporangia per field (minimum of 10 randomly chosen microscopic fields, x40 objective magnification) and culturing 10-fold diluted homogenates on Sabouraud dextrose agar. Data are means ± SEM (*n* = 4/group). Ns, not significant.

Mammary infection with *P. bovis* was confirmed microscopically in *Camp*^+/+^ and *Camp*^−/−^ mice 4 days post-infection. Using PAS and GMS staining, round to oval sporangia with internal divisions compatible with *Prototheca* spp. were visible both free within alveolar lumen and throughout the mammary gland interstitium (Figure 2A). The grade of infection with *P. bovis* did not differ between *Camp*^+/+^ and *Camp*^−/−^ mice at 4 days post-infection, as determined by histological counting of sporangia and *Prototheca* spp. culture (Figure 2B).

**Figure 3:**
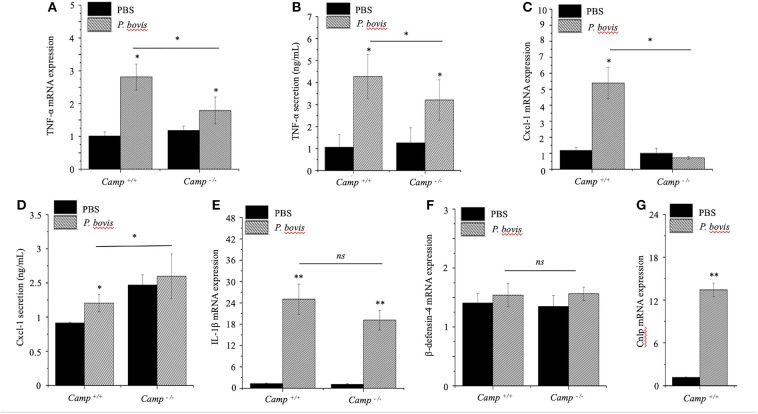
Lesser synthesis of pro-inflammatory cytokines in *Camp*^−/−^ mice infected with *Prototheca bovis*. *Camp*^+/+^ and *Camp*^−/−^ were intramammary challenged with *P. bovis* or PBS (control). Transcriptomic expression and protein secretion of pro-inflammatory genes in the mammary gland including **(A,B)** *TNF-*α, **(C,D)** *Cxcl-1*, **(E)** *IL-1*β, **(F)** β*-defensin-*4, and **(G)** *Camp* were determined at 4 d post-infection. mRNA synthesis was quantified using RT qPCR and protein secretion was determined in cell supernatant by ELISA. Data are means ± SEM (*n* = 4/group). *p < 0.05, **p < 0.01 (one-way ANOVA *post hoc* Bonferroni correction) was considered significant.

The general process of mastitis is characterized by upregulated synthesis of certain pro-inflammatory cytokines, such as *TNF-*α and *IL-1*β (Bannerman et al., 2004). *P. bovis* induced mRNA synthesis and protein secretion of TNF-α in mammary glands from both *Camp*^+/+^ and *Camp*^−/−^ mice infected with *P. bovis*, with the highest concentration in *Camp*^+/+^ mice (*p* < 0.05, Figures 3A,B).

Cxcl-1 chemoattracts leukocytes to sites of infection (De Filippo et al., 2013); recruitment of neutrophils and monocytes/macrophages from blood to milk compartments is a critical defense mechanism in bovine mastitis (Zhang et al., 2012). In our study, *Cxcl-1* mRNA was upregulated in *P. bovis* infected mammary glands of *Camp*^+/+^ relative to *Camp*^−/−^ mice (*p* < 0.05, Figure 3C). Secretion of Cxcl-1 increased in mammary glands from both *Camp*^+/+^ and *Camp*^−/−^ infected mice relative to non-infected controls but, the highest level was in *Camp*^−/−^ infected mammary glands (*p* < 0.05, Figure 3D). Transcriptional *IL-1*β gene levels were increased after *P. bovis* challenge, but not different between *Camp*^+/+^ and *Camp*^−/−^ infected mammary glands (*p* > 0.05, Figure 3E). Transcriptomic gene expression of host defense peptides in mammary tissue revealed that β-defensin-4 was not increased after *P. bovis* infection in mammary glands of *Camp*^−/−^ and *Camp*^+/+^ mice (*p* > 0.05, Figure 3F), whereas *Camp* mRNA was increased in infected mammary glands of *Camp*^+/+^ mice compared to uninfected *Camp*^+/+^ mice (*p* < 0.05, Figure 3G). No *Camp* gene expression was detected in *Camp*^−/−^ mice (data not shown). Taken together, *P. bovis* provoked acute mastitis in experimentally challenged mice. Furthermore, endogenous production of cathelicidins, induced by the algae, was associated with increased synthesis of pro-inflammatory *TNF-*α and *Cxcl-1*.

### Bone Marrow-Derived Macrophages Deficient in Cathelicidin (Camp^−/−^) Had Impaired Pro-inflammatory Cytokine Response to *P. bovis* Infection

**Figure 4:**
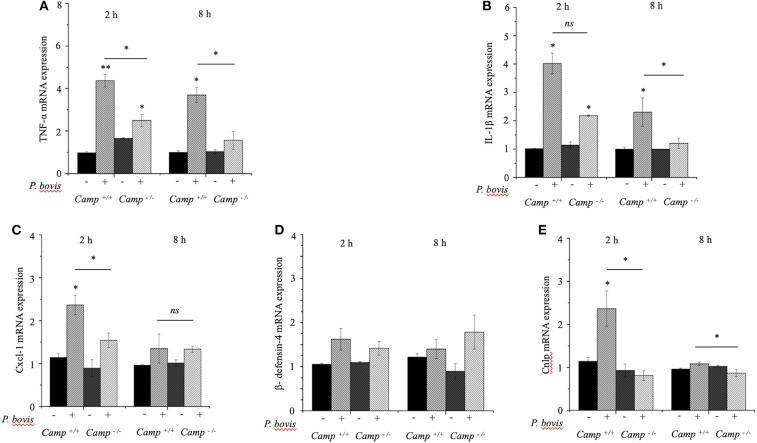
Reduced production of pro-inflammatory cytokines in macrophages from *Camp*^−/−^ exposed to *Prototheca bovis*. **(A)** Primary bone marrow-derived macrophages were isolated from *Camp*^+/+^ and *Camp*^−/−^ mice and challenged with *P. bovis*. **(A,B)** Transcriptomic expression of **(A)** *TNF-*α, **(B)** *IL-1*β, **(C)** *Cxcl-1*, **(D)** β*-defensin-4*, and **(E)** *Camp* was determined at 2 and 8 h post-infection. mRNA synthesis was quantified using RT qPCR. Data are means ± SEM (*n* = 3 independent experiments done in triplicate). *p < 0.05, **p < 0.01 (one-way ANOVA *post hoc* Bonferroni correction) was considered significant. Ns, not significant.

Macrophages prevailed in mastitis induced by *P. bovis* in mice (Figure 1D). Furthermore, when exposed to inflammatory stimuli (e.g., *Prototheca* spp), macrophages secrete a vast array of pro-inflammatory cytokines (Arango Duque and Descoteaux, 2014). To determine if cathelicidins modulate macrophage function in protothecal mastitis, BMDMs from *Camp*^+/+^ and *Camp*^−/−^ mice were challenged with *P. bovis*. *P. bovis* induced early (2 h) *TNF-*α and *IL-1*β transcription in BMDMs, with higher levels in *Camp*^+/+^ BMDMs (*p* < 0.05) (Figures 4A,B). Such *TNF-*α and *IL-1*β overexpression lasted for at least 8 h post-infection (Figures 4A,B). *Prototheca bovis* also induced early (2 h) increased *Cxcl-1* mRNA expression in *Camp*^+/+^ BMDMs compared to *Camp*^−/−^ BMDMs (*p* < 0.05, Figure 4C). *Prototheca bovis* did not induce β*-defensin-4* mRNA (*p* > 0.05, Figure 4D) whereas *P. bovis* increased *Camp* gene expression in *Camp*^+/+^ BMDMs with an expected non-expression in *Camp*^−/−^ BMDMs (*p* < 0.05, Figure 4E). Thus, endogenous cathelicidin contributed to production of pro-inflammatory cytokines in macrophages exposed to *P. bovis*.

### Human Cathelicidin LL-37 Inhibited Growth of *P. bovis* and Decreased Pro-inflammatory Responses in Infected Macrophages and Mammary Epithelial Cells

**Figure 5:**
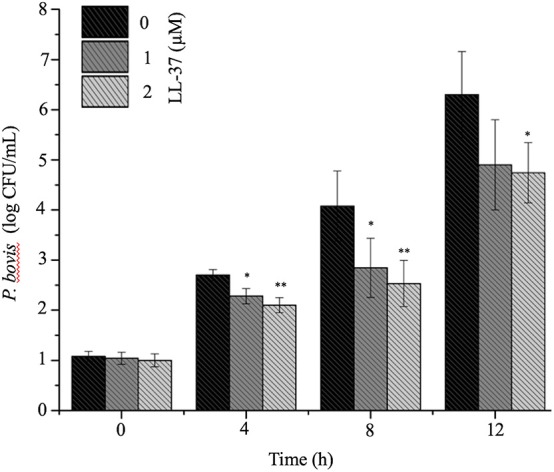
Synthetic human cathelicidin LL-37 inhibited *in vitro Prototheca bovis*. *Prototheca bovis* (1 × 10^5^ CFU/mL in logarithmic growth phase) was incubated with synthetic human cathelicidin LL-37 peptides (up to 2 μM for up 12 h at 37°C) into Sabouraud dextrose broth and algae concentration were determined using a standard curve, with only *P. bovis* and expressed as log CFU/mL. Histogram of remaining *P. bovis* after peptide treatment. Data are shown as means ± SEM (*n* = 3 independent experiments done in triplicate). *p < 0.05, **p < 0.01 (one-way ANOVA *post hoc* Bonferroni correction) was considered significant.

**Figure 6:**
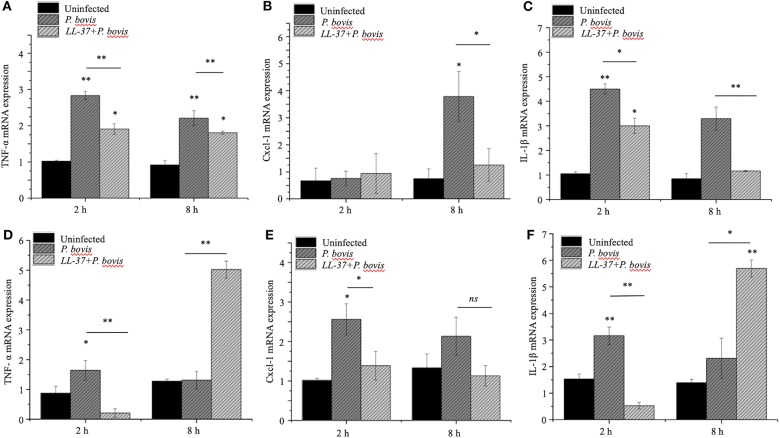
Synthetic human cathelicidin LL-37 mitigated production of pro-inflammatory cytokines in murine macrophages and mammary epithelia infected with *Prototheca bovis*. Murine phagocytic (J774.A1) macrophages **(A–C)** and mammary epithelial (HC-11) cells **(D–F)** were challenged with *P. bovis* (1 × 10^5^ cfu/mL) ± synthetic human cathelicidin LL-37 (2 μM) (37°C with 5% CO_2_). Transcriptomic expression of **(A,D)** *TNF-*α, **(B,E)** *Cxcl-1* and **(C,F)** *IL-1*β were determined at 2 and 8 h post-infection. mRNA synthesis was quantified using RT qPCR. Data are shown as means ± SEM (*n* = 3 independent experiments done in triplicate). *p < 0.05, **p < 0.01 (one-way ANOVA *post hoc* Bonferroni correction) was considered significant. Ns, not significant.

To mechanistically explore the role of cathelicidin in *P. bovis* mastitis, we studied whether exogenous cathelicidin exert algicidal or immunomodulatory effects in key cellular components in mastitis: macrophages and mammary epithelium. In regard to direct killing, synthetic human cathelicidin LL-37 (1 and 2 μM) decreased *in vitro* growth of *P. bovis* up to 8 h, with some inhibitory activity still present at 24 h (*p* < 0.05, Figure 5). In reference to the immunomodulatory role of LL-37, *P. bovis* upregulated mRNA expression of *TNF-*α, *Cxcl-1*, and *IL-1*β in murine phagocytic J774.A1 cells (at 2 and 8 h post-infection), whereas co-stimulation with LL-37 decreased concentrations of *TNF-*α, *Cxcl-1*, and *IL-1*β mRNA expression (*p* < 0.05, Figures 6A–C). Next, the immunoregulatory role of LL-37 was assessed in murine mammary epithelial cells (HC11) capable of producing milk casein protein. Challenge of HC11 cells with *P. bovis* induced early (2 h) *TNF-*α, *Cxcl-1* and *IL-1*β gene expression (*p* < 0.05, Figures 6D–F), whereas co-stimulation with LL-37 lowered these mRNA *TNF-*α, *Cxcl-1*, and *IL-1*β responses. Addition of LL-37 to the mammary epithelium stimulated transcriptional expression of *IL-1*β and *TNF-*α at a later point (8 h post-infection) (*p* < 0.05, Figures 6D–F). Thus, human cathelicidin LL-37 reduced synthesis of pro-inflammatory cytokines in murine macrophages and mammary epithelium, whereas it had some direct killing effects on *P. bovis*.

## Discussion

Although *P. bovis* is a major cause of insidious and chronic (>30 days) mastitis in cattle (Moller et al., 2007; Wawron et al., 2013; Bozzo et al., 2014; Jagielski et al., 2019b), the udder innate immune response to pathogenic *Prototheca* spp. remains poorly understood. In this study, *P. bovis* triggered severe acute mastitis in mice and naturally occurring cathelicidins aided in establishing local inflammation by promoting synthesis of pro-inflammatory cytokines. Two main lineages of *Prototheca* spp. have been relevant in public health: a dominant species typically associated with dairy cattle, namely *P. ciferrii* (formerly *P. zopfii* GT-I), *P. blaschkeae*, and *P. bovis* (formerly *P. zopfii* GT-II) and others human-associated *Prototheca* spp. (i.e., *P. wickerhamii, P. cutis, P. miyajii*) (Jagielski et al., 2019a). In this study, we used a *Prototheca* spp. identified as *P. bovis* following a taxonomic approach commonly accepted for Prototheca (Roesler et al., 2006) and a cytb-based genotyping used for unambiguous *Prototheca* spp. identification (Jagielski et al., 2018) based on the protothecal phylogeny (Jagielski et al., 2019a).

Although *Camp*^−/−^ mice had increased infiltration of macrophages in infected mammary glands, they also produced less local expression and secretion of TNF-α and Cxcl-1. Moreover, BMDMs isolated from *Camp*^−/−^ mice had lower *in vitro* synthesis of pro-inflammatory TNF-α, IL-1β, and Cxcl-1 in response to *P. bovis*. That Cxcl-1 secretion in infected *Camp*^−/−^ mice ended up higher than in wild type mice denoted a lack of association between Cxcl-1 mRNA and protein kinetics. Such differences in cytokine mRNA/protein expressions are usually due to post-transcriptional mechanisms (Greenbaum et al., 2003) or perhaps, *Cxcl*-1 gene transcription is augmented only at early points by cathelicidins or the *Cxcl*-1 protein half-life is longer compared with mRNA. Alternatively, increased Cxcl-1 in infected *Camp*^−/−^ mice may correspond to an overwhelming and generalized inflammation, higher than any cathelicidin effect in the wild type. Overall, the observed role of endogenous cathelicidin promoting *Cxcl*-1 in protothecal mastitis could be critical in attracting leukocytes early to sites of infection and retain macrophages into the udder during the innate immune response against pathogenic algae. In agreement, murine cathelicidin Cramp attracted *in vitro* T-cells, macrophages, neutrophils, eosinophils and mast cells via an FPRL-1 receptor (Yang et al., 2000; Kurosaka et al., 2005; Tjabringa et al., 2006) whereas LL-37 chemoattracted leukocytes via G-protein coupled receptors (Vandamme et al., 2012). Cathelicidins could also chemoattract neutrophils indirectly, by inducing chemokines. In this regard, cathelicidins induced CXCL8 transcription alone and in synergy with TNFα though *Src* family kinase pathways downstream of P2X7R in keratinocytes (Nijnik et al., 2012; Chen et al., 2013) and gingival fibroblasts (Montreekachon et al., 2011) and in an EGFR-dependent manner in skin (Wu et al., 2010) and airway epithelial cells (Tjabringa et al., 2006). Whereas, the influx of leukocytes in the mammary gland during *P. bovis* infection could be functionally regulated by cathelicidin, other cathelicidin-independent chemoattractive effects cannot be disregarded. For instance, cell-surface glycoproteins involved in cell-cell interaction, such as CD44, which mediates specific adhesion of bovine blood polymorphonuclears to mammary epithelial cells (Gonen et al., 2008), could initiate recruitment of inflammatory cells during mastitis.

Our study demonstrated that a cathelicidin can exert an immunosuppressive effect in another species; in particular, human LL-37 mitigated production of pro-inflammatory TNF-α, IL-1β, and Cxcl-1 in murine macrophages and mammary epithelial cells challenged with *P. bovis*. This immunomodulatory role of human cathelicidin LL-37 was not surprising, as LL-37 reduced secretion of IL-1β, IL-6, IL-8, and TNF-α in human and murine neutrophils exposed to heat-inactivated *Pseudomonas aeruginosa* or *Staphylococcus aureus* (Alalwani et al., 2010). Likewise, human or murine macrophage-like cells had reduced expression of TNF-α and nitric oxide (NO) in the presence of LL-37 when stimulated with bacterial lipooligosaccharide or LPS (Scott et al., 2002; Zughaier et al., 2005). Mechanistically, LL-37 could directly bind LPS in macrophages, thereby suppressing IL-6, IL-1β, and TNF-α synthesis (Nagaoka et al., 2001; Tomasinsig et al., 2010).

In terms of algicidal effects, there was no differences between *Camp*^+/+^ and *Camp*^−/−^ infected mice in *P. bovis* burden in mammary glands at an early point of infection (4 days). In contrast, human cathelicidin LL-37 *in vitro* directly reduced number of *P. bovis* in a dose-dependent manner. These killing properties of cathelicidins, LL-37 in this case, are related with the cationic peptide capacity to bind and disrupt negatively charged microbial membranes, leading to cell death due to destabilization of plasma membranes and efflux of ATP and proteins (Tsai et al., 2014). Cathelicidins can also cross membranes and disrupt intracellular processes, including RNA and DNA synthesis and protein degradation (Mansour et al., 2014). Although the role of cathelicidins in fungal/algal infectious is not fully explored, there is increasing evidence of protective effects. Human LL-37 has anti-fungus and algae activity against *Candida albicans, Malassezia furfur, Trichophyton rubrum* and *Trichophyton. mentagrophytes* (Lopez-Garcia et al., 2006; Tomasinsig et al., 2012; Tsai et al., 2014). Cattle have a vast repertoire of cathelicidins, with at least 8 naturally occurring (Young-Speirs et al., 2018) and direct killing effects on *P. bovis* observed in cathelicidins from cattle origin. Bovine myeloid antimicrobial peptide (BMAP)-28 displayed quick anti-*Prototheca* activity (<1 h), with extensive surface blebbing and release of intracellular material due to cell permeabilization (Tomasinsig et al., 2012) whereas other bovine host defense peptides (bactericin 5 and lingual anticrobial peptide; LAP) also had anti *Prototheca* activity, albeit through non-lytic mechanisms (Tomasinsig et al., 2012). Conversely, a different susceptibility of *P. bovis* to cathelicidins of diverse origins agrees with studies in bacteria. Human LL-37 and murine Cramp had dissimilar minimum inhibitory concentrations (MICs) against *Citrobacter rodentium, Pseudomonas aeruginosa, Streptococcus pyogenes, Staphylococcus aureus, Escherichia coli, Salmonella* spp. and *Helicobacter pylori* (Iimura et al., 2005; Coorens et al., 2017; Marin et al., 2019). In addition, endogenous Cramp had a pro-inflammatory role in murine mastitis induced by *P. bovis*, whereas in contrast, LL-37 reduced synthesis of pro-inflammatory cytokines in macrophages and mammary epithelia. Although human LL-37 and murine Cramp are considered homologous based on broadly similar and interspecies functions (antimicrobial and immunomodulatory) (Barlow et al., 2011) and structural properties (α-helical and net charge of +6), they actually share <70% sequence identity in their active peptide (Nizet et al., 2001; Tomasinsig and Zanetti, 2005). This may explain singular activities for each peptide and indeed, human LL-37 binded dsRNA and activated TLR3 signaling in early endosomes but murine Cramp did not (Singh et al., 2013). Likewise, LL-37 was more effective in reducing *S. aureus* proliferation *in vitro* when compared to Cramp (Coorens et al., 2017). In our study, murine cathelicidin Cramp may not have killing properties against *P. bovis* or perhaps acts later on the pathogen burden, when immune defenses are developed and more Cramp is available. Alternatively, Cramp may have indirect antimicrobial effects, promoting neutrophil killing by increasing production of reactive oxygen species (ROS) and enhancing phagocytic activity (Alalwani et al., 2010). In all cases, cathelicidin LL 37 appeared to be a good model of cathelicidins and potential target molecule for developing therapeutics, although comparative studies with various cathelicidins and their derivatives may offer distinctive immunomodulatory advantages.

Mast cells in mammary glands infected with *P. bovis* were an unexpected finding. Mast cells are immune effectors that degranulate pro-inflammatory granules rich in histamine and heparin upon activation. LL-37 stimulated degranulation of mast cells (Niyonsaba et al., 2010) through MAP kinases p38 and ERK phosphorylation, increasing vascular permeability in skin (Chen et al., 2006). Thus, such release of histamine, proteoglycans, serotonin and serine proteases from degranulated mast cells, likely regulated by cathelicidin, may be important in pathogenesis of *P. bovis* and other mastitis pathogens (e.g., *Staphyloccocus aureus, Pseudomonas*) by augmenting inflammation (e.g., increasing permeability of capillaries to permit leukocyte extravasation).

In summary, pathogenesis of *P. bovis*, a globally ubiquitous and environmental (grass, water, trees) algae (Rösler et al., 2003) capable of infecting various vertebrate hosts (Seok et al., 2013; Capra et al., 2014; Carfora et al., 2017) remains poorly understood. Therapeutic control for human and animal protothecosis is on demand. Whereas, a human cathelicidin (LL-37) could kill *P. bovis*, endogenous cathelicidin (Cramp) contributed to the inflammatory response during *P. bovis*-induced mastitis, likely recruiting early leukocytes, thereby aiding pathogen control. Whereas, we focused on early steps in protothecal mastitis, cathelicidins could have other effects through later stages of inflammation due to known interactions with many cell types, including endothelial cells (Koczulla et al., 2003), epithelial cells (Tjabringa et al., 2003), mast cells (Niyonsaba et al., 2001) and macrophages (Scott et al., 2002). Cathelicidins were pleitrophic, either preventing (Kirikae et al., 1998) or augmenting inflammatory reactions, e.g., skin with rosacea (Yamasaki et al., 2007). Therefore, this study represented a proof-of-concept regarding the role of innate immune responses contributed by cathelicidins in protothecal mastitis; therefore, we propose LL-37 has potential as a therapeutic compound for controlling pathogenic algae. More studies are needed to decipher the kinetics of myeloid and non-myeloid cells and cytokine milieu that regulate specific functions of cathelicidins in mastitis.

## Data Availability Statement

The datasets generated for this study are available on request to the corresponding author.

## Ethics Statement

The animal study was reviewed and approved by Animal experiments were conducted in accordance with Canadian Guidelines for Animal Welfare (CGAW) and the University of Calgary Animal Care Committee.

## Author Contributions

MS, PC, and JG performed the *in vitro* and *in vivo* experiments. CK conducted the histopathologic analysis. MS and JG performed the analysis of data and prepared the figures. MS, BH, HB, and EC conceived this research, designed the experiments, and wrote the manuscript. All authors reviewed the manuscript.

### Conflict of Interest

The authors declare that the research was conducted in the absence of any commercial or financial relationships that could be construed as a potential conflict of interest.

## Supplementary Material

Supplementary Figure 1 Cytochrome b (cytb) PCR and PCR-RFLP for identification of the *Prototheca bovis*. The *Prototheca spp*. isolate recovered from the milk of a cow with clinical mastitis and used in our murine mastitis model was genotyped by cytb PCR and PCR-RFLP in comparison with a *P. ciferri* reference strain isolated from the enviroment. **(A)** Cytb gene PCR results depicting a 644 bp PCR product for *P. ciferri* strain (Lane 1) and the *P. bovis* isolated from the milk of a cow with clinical mastitis and used in our murine model of mastitis (Lane 2). DNA ladder: molecular-weight size marker. **(B)** Cytb PCR-RFLP results showing the *P. ciferri* given 300 and 350 bp DNA fragments (from the 644 bp PCR product) after TaiI digestion (Lane 1) and the *P. bovis* isolated from the milk of a cow with clinical mastitis given DNA fragments of 200 and 450 bp (from the 644 bp PCR product) after digestion with TaiI, compatible with *P. bovis* (Lane 2). DNA ladder: molecular-weight size marker.
