# Supplementary figures and images for: Murine and Human Cathelicidins Contribute Differently to Hallmarks of Mastitis Induced by Pathogenic Prototheca bovis Algae
Source: Front Cell Infect Microbiol. 2020 Feb 7;10:31. doi: 10.3389/fcimb.2020.00031 (PMC7025567; doi:10.3389/fcimb.2020.00031)

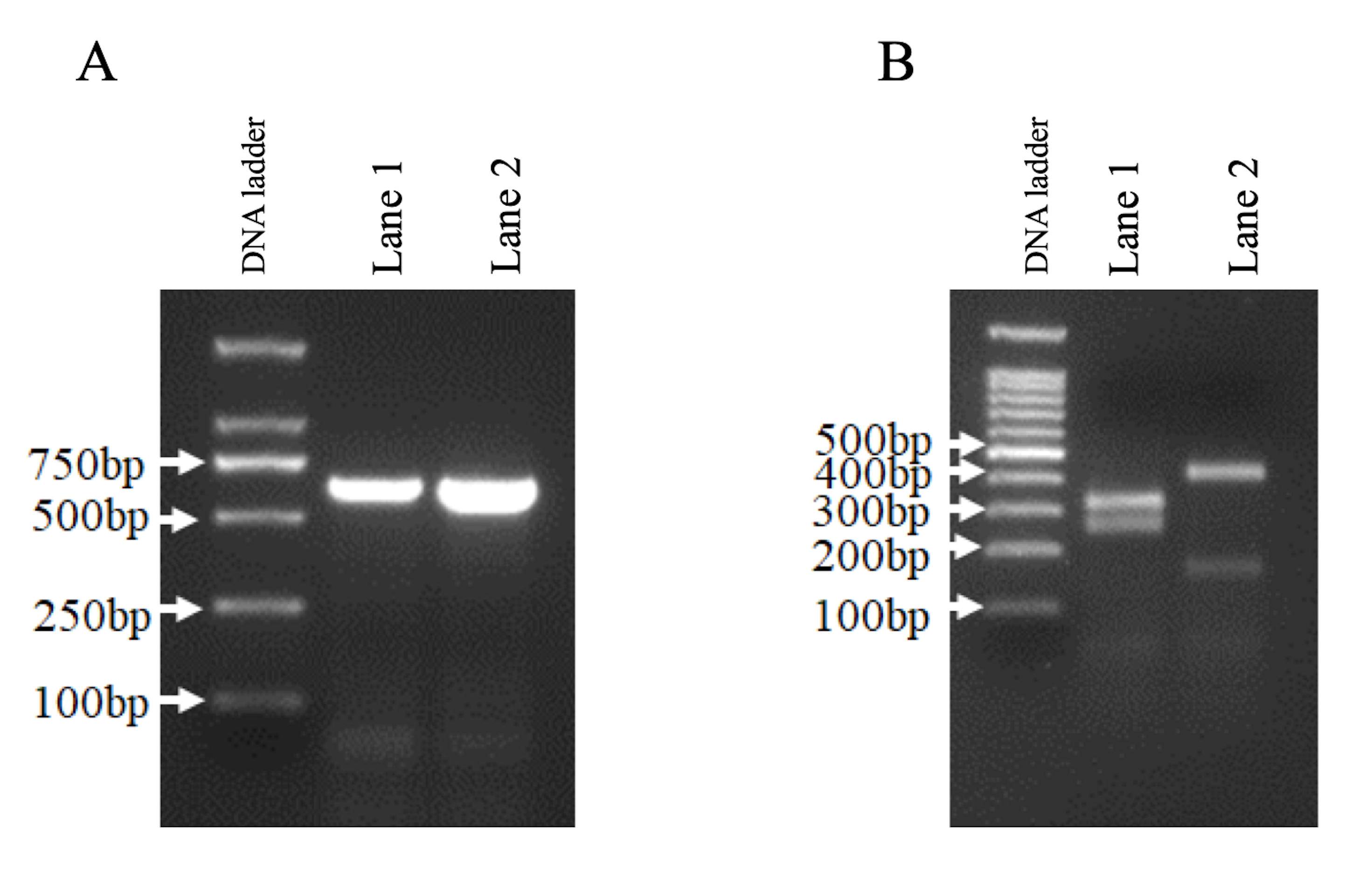

Supplement: Supplementary Figure 1 — Cytochrome b (cytb) PCR and PCR-RFLP for identification of the Prototheca bovis. The Prototheca spp. isolate recovered from the milk of a cow with clinical mastitis and used in our murine mastitis model was genotyped by cytb PCR and PCR-RFLP in comparison with a P. ciferri reference strain isolated from the enviroment. (A) Cytb gene PCR results depicting a 644 bp PCR product for P. ciferri strain (Lane 1) and the P. bovis isolated from the milk of a cow with clinical mastitis and used in our murine model of mastitis (Lane 2). DNA ladder: molecular-weight size marker. (B) Cytb PCR-RFLP results showing the P. ciferri given 300 and 350 bp DNA fragments (from the 644 bp PCR product) after TaiI digestion (Lane 1) and the P. bovis isolated from the milk of a cow with clinical mastitis given DNA fragments of 200 and 450 bp (from the 644 bp PCR product) after digestion with TaiI, compatible with P. bovis (Lane 2). DNA ladder: molecular-weight size marker. [file Image_1.TIFF]
